# Bone marrow suppression as a complication of total skin helical tomotherapy in the treatment of mycosis fungoides

**DOI:** 10.1186/s13014-018-1013-2

**Published:** 2018-04-13

**Authors:** Eric M. Schaff, Stephen A. Rosenberg, Stephanie J. Olson, Steven P. Howard, Kristin A. Bradley

**Affiliations:** 1 Michigan State University College of Human Medicine, 418 W. Magnetic Street, Marquette, MI 49855 USA; 2 Department of Human Oncology, University of Wisconsin, Madison, WI USA

**Keywords:** Mycosis fungoides, Helical tomotherapy, Total skin electron beam therapy, Cutaneous lymphoma, T-cell lymphoma

## Abstract

**Background:**

Total skin electron beam therapy (TSEBT) is an effective treatment in mycosis fungoides. Total skin helical tomotherapy (TSHT) may be an alternative to TSEBT and may offer several dosimetric and treatment advantages. There are currently very few published treatment results using TSHT in place of TSEBT for treatment of mycosis fungoides.

**Case presentation:**

Two patients with mycosis fungoides were treated at our institution using TSHT. The first patient was a 69-year-old Caucasian female with stage IVA2 (T2 N3 M0 B2) disease who was treated to a dose of 12 Gy in 8 fractions, with a bone marrow mean dose of 1.66 Gy and V10 = 0.41%. Two weeks after ending treatment the patient developed myelosuppression including grade 4 thrombocytopenia and required blood and platelet transfusions. The second patient was a 29-year-old Caucasian female with stage I (T2 N0 M0 B0) disease. This patient previously had been treated for mycosis fungoides using helical tomotherapy (HT) at a dose of 20 Gy to a localized region and experienced mild thrombocytopenia at that time. The patient then underwent retreatment 17 months later with TSHT to a dose of 12 Gy in 6 fractions with a mean bone marrow dose of 2.3 Gy and V10 = 4.28%. This patient once again experienced myelosuppression that included grade 4 thrombocytopenia. She also required blood and platelet transfusions.

**Conclusions:**

Both patients treated with TSHT experienced severe bone marrow suppression including grade 4 thrombocytopenia. This was more severe than expected considering the relatively low overall prescription dose and despite a planning constraint placed on the bone marrow of a mean dose of < 2 Gy. These outcomes suggest that patients treated using TSHT should be closely monitored for myelosuppression and caution used even when treating to a dose of 12 Gy.

## Background

TSEBT has been proven to be an effective treatment in T2 and T3 mycosis fungoides that is refractory to other treatment modalities. Although it has been proven effective, TSEBT has disadvantages that may complicate treatment [1]. The most common protocol used for treatment is the Stanford Protocol that requires the patient to stand for the entirety of treatment that can last 30 min [2]. Total skin electron therapy may be limited secondary to heterogeneity with areas receiving up to 40% deviation from the prescription dose [3]. There have been alternative protocols proposed which allow for the patient to lie down but these protocols sacrifice dose homogeneity to an even greater extent [4].

Due to its dose delivery characteristics, helical tomotherapy (HT) with photons can be used to treat superficially and thus may be an alternative to TSEBT. Treating patients with total skin helical tomotherapy (TSHT) may provide several advantages: 1) improved dose homogeneity; 2) non-overlapping fields; and 3) patients in a comfortable supine position for treatment.

Although there are potential benefits to the use of TSHT, there remains concern that photon therapy may lead to higher bone marrow doses and potential toxicity compared to TSEBT. Recent data regarding TSEBT suggest that lower doses of radiation may provide equally effective control of the disease, less toxicity, and leave the possibility for retreatment if relapse occurs [5]. A pooled analysis of 3 phase II trials showed an overall response of 88% and complete response of 27% treating with 12 Gy utilizing TSEBT [6]. Only 3% of patients experienced grade 1 leukopenia in these trials. If lower doses are equally effective using TSHT as well, it is possible that the treatment toxicity can be spared while still maintaining treatment efficacy with a photon-based approach. Herein we describe the outcomes of 2 patients treated with TSHT and the toxicity associated with treatment.

## Case presentation

**Fig. 1:**
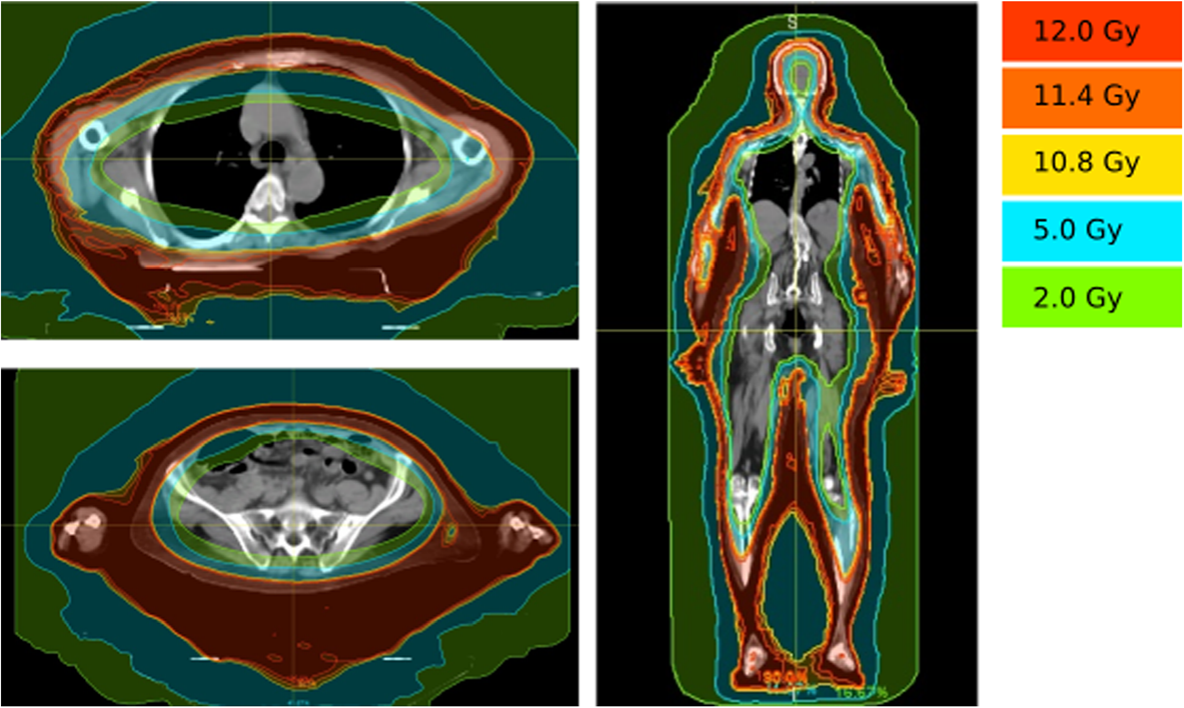
Dose distributions for patient 1 treated with TSHT for mycosis fungoides

**Fig. 2:**
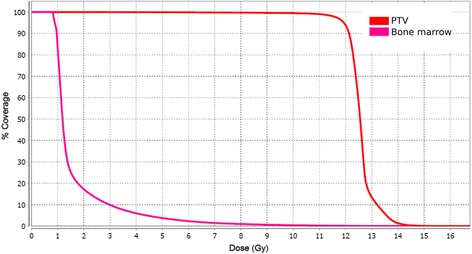
Dose-volume histogram for patient 1 treated with TSHT for mycosis fungoides

The first patient was a 69-year-old Caucasian female with stage IVA2 (T2 N3 M0 B2) mycosis fungoides who presented to the radiation oncology clinic for treatment after failing interferon alfa-2b, photopheresis, vorinostat, brentuximab vedotin, and gemcitabine. She was treated by our department using TSHT to a dose of 12 Gy in 8 fractions 4 days per week. The patient underwent simulation and treatment with a head and shoulder mask, body fix, with arms down. The dose distribution and dose-volume histogram are shown in Figs. 1 and 2. Bone marrow mean dose was 1.66 Gy with V10 = 0.41%. The bone marrow contour sum included the entire spine, pelvis and femurs. Dose values to other bony sites have been included for additional reference in Table 1.

**Table 1:** Patient 1 dose values

	Mean	V10	V5	V3
Bone marrow total (arms not included)	1.66 Gy	0.41%	3.72%	9.81%
Bone marrow total (arms included)	2.62 Gy	7.97%	13.45%	19.03%
Lumbar	1.51 Gy	0.97%	3.15%	7.65%
Dorsal	1.95 Gy	0.49%	3.76%	11.19%
Pelvic	1.49 Gy	0.32%	3.16%	7.55%
Femurs	1.86 Gy	0.12%	4.86%	14.09%
Arms	11.18 Gy	74.54%	98.84%	100%

On the last day of treatment, she developed grade 1 anemia and thrombocytopenia. Two weeks following the last treatment the bone marrow toxicity progressed to grade 2 anemia, grade 2 leukopenia, and grade 4 thrombocytopenia. The patient was given multiple transfusions of packed red blood cells (PRBCs) and platelets over the course of treatment. One month after treatment she experienced an episode of epistaxis that self-resolved after 40 min. Within 1 week of treatment completion, the patient noted significant improvement of her diffuse erythroderma. Despite radiotherapy she had progressive disease on her right thigh several weeks later and began treatment with chlorambucil and low dose prednisone. She continued chlorambucil until she was hospitalized and ultimately passed away secondary to stroke several months after treatment.

The second patient was a 29-year-old Caucasian female with stage I (T2 N0 M0 B0) mycosis fungoides. She had previous treatment with topical steroids, methotrexate, interferon, mycophenolate, and cyclosporine. She was on photochemotherapy with interferon alfa-2b at the time of presentation. At that time, she was treated with photons to active sites of disease with a dose of 20 Gy in 10 fractions to the scalp, buttocks, neck, and axillae, and a dose of 10 Gy to the back in 5 fractions. The patient experienced improvement in disease burden. However, the patient also experienced myelosuppression beginning 2 weeks after this treatment with grade 3 leukopenia, grade 1 anemia, and grade 4 thrombocytopenia**.**

**Fig. 3:**
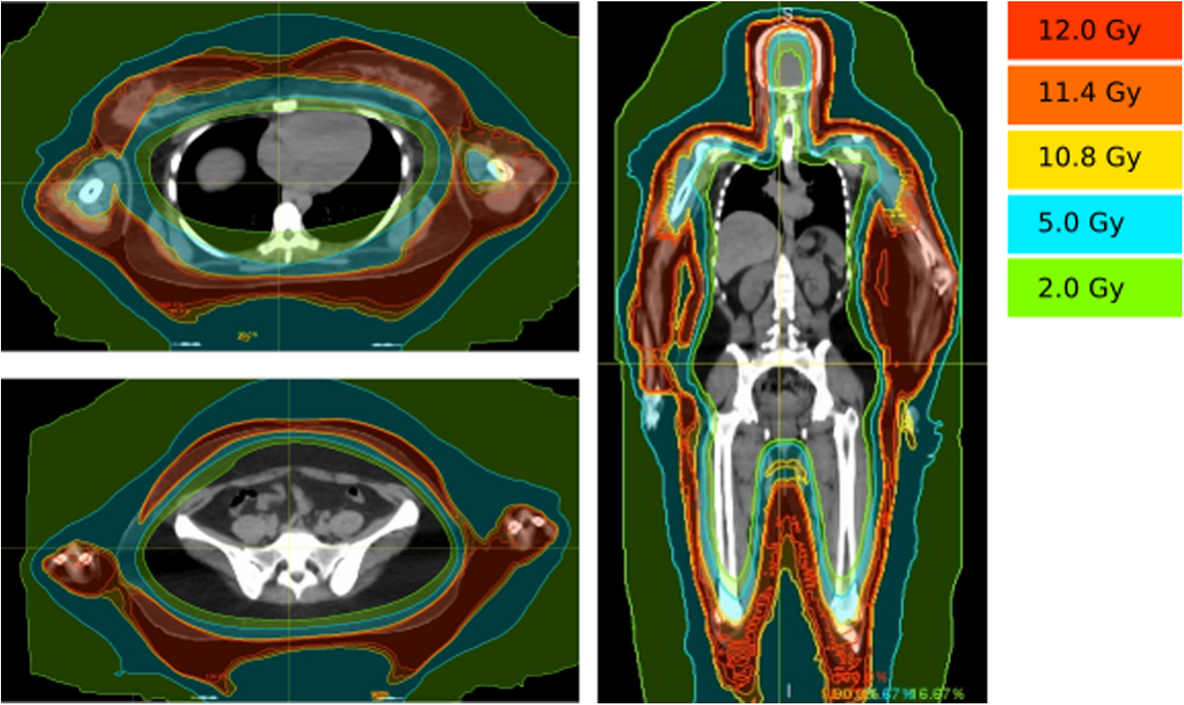
Dose distributions for patient 2 treated with TSHT for mycosis fungoides. Note that the hands and feet of this patient were spared treatment

**Fig. 4:**
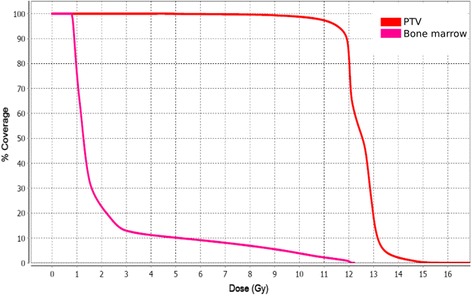
Dose-volume histogram for patient 2 treated with TSHT for mycosis fungoides

She then presented again to the radiation oncology clinic after progression of disease approximately 17 months from her previous radiotherapy treatment. She was being treated with interferon alfa-2 and methotrexate. She was then treated to a dose of 12 Gy in 6 fractions with daily treatments using TSHT with sparing of the hands and feet. The patient underwent simulation and treatment with a facemask and body fix with arms down The upper half and lower half of the body were treated separately with each hemibody receiving 6 fractions of 2 Gy. The dose distribution and dose-volume histogram are shown in Figs. 3 and 4. Bone marrow mean dose was 2.3 Gy with V10 = 4.28%. As with the first patient, the bone marrow contour sum includes the entire spine, pelvis and femurs. Dose values to other bony sites have been included for additional reference in Table 2.

**Table 2:** Patient 2 dose values

	Mean	V10	V5	V3
Bone marrow total (arms not included)	2.3 Gy	4.28%	10.11%	12.93%
Bone marrow total (arms included)	3.56 Gy	13.76%	24.12%	27.49%
Lumbar	1.18 Gy	0	0	0.76%
Dorsal	1.87 Gy	0	0.12%	2.61%
Pelvic	1.13 Gy	0	0.03%	0.71%
Femurs	4.53 Gy	14.31%	37.38%	44.77%
Arms	10.41 Gy	62.97%	93.77%	100%

Initially the patient had a good response to therapy with many of the cutaneous lesions resolving, however her disease rapidly progressed 2 months following treatment. Bone marrow suppression occurred 3 weeks after treatment with grade 1 anemia, grade 3 leukopenia, and grade 4 thrombocytopenia. The patient did not report any bleeding events, however red blood cell and platelet transfusions were necessary before the patient’s myelosuppression eventually resolved 6 months after the end of treatment.

## Discussion and conclusions

Both patients treated with TSHT at our institution experienced significant bone marrow toxicity. Even at a prescription dose of 12 Gy, these patients experienced grade 4 myelosuppression. Their platelet nadir occurred 2 and 3 weeks after treatment ended. This was similar to the 2-week platelet nadir Carabell et al. experienced in patients treated with total body irradiation (TBI) to a dose 1.5 Gy for advanced non-Hodgkin lymphoma [7]. Similar bone marrow toxicities have been reported when using TBI to a dose of 1.5 Gy with concurrent lonidamine to treat patients with favorable B-cell neoplasms as well [8]. Our results show similar rates of myelosuppression as patients treated using TSHT at a higher prescription dose of 30 Gy in the series by Hsieh et al. [9]. However, in the prior report, the mean bone marrow dose varied between 4 to 9 Gy. In our two patients, the mean bone marrow doses were significantly lower at 2.3 Gy and 1.66 Gy.

In a Japanese case series, 3 patients were treated for mycosis fungoides using TSHT to a dose of 10 Gy and myelosuppression occurred in 2 patients [10]. One of the patients experienced grade 4 myelosuppression which later required blood transfusion. This study is comparable to our own, as prescription doses (12 Gy vs 10 Gy) were similar. In addition, their mean bone marrow dose of 2.27 Gy was comparable to our patients. Fractionation similar to our first patient (12 Gy in 8 fractions) has been shown to result in rare grade 2 and grade 3 toxicities without myelosuppression when using TSEBT instead of TSHT [11, 12].

The question remains as to why our patients and the patient from the previously mentioned case study experienced severe myelosuppression with such low mean bone marrow dose. One explanation is that our treatment planning software may have not have accurately simulated the actual bone marrow dosage. Another possibility is that the planning parameter of a mean bone marrow dose < 2 Gy is not a strict or predictive enough treatment planning constraint for total skin treatments. Thrombocytopenia experienced in these two patients may be secondary to significant volume of low dose irradiation to the bone marrow. A total body exposure of 2.5–5 Gy results in hematopoietic syndrome. This occurs secondary to destruction of mitotically active precursor cells approximated 3–4 weeks after exposure. In these patients, mean doses to the bone marrow did approach 2 Gy and likely led to similar biological effects and the resultant thrombocytopenia.

One method that may decrease internal dose would be using a bolus to bring the dose closer to the skin surface. This was done in an anthropomorphic phantom substitute study by Lin et al. in which TSHT was used to produce a dose of at least 26 Gy to a depth of 4 mm of the truncal skin in conjunction with a 3 mm neoprene diving suit utilized as bolus [13]. This plan resulted in a simulated mean dose to OARs of 9.7 cGy per fraction during the 36 Gy treatment course of 36 fractions as compared to approximately 1–4 cGy/fx with a rival TSEBT plan. However, they defined the OARs to be heart, lung, liver, kidney, spleen, intestine, and rectum, while bone marrow was not included. Hsieh et al. also used a 3 mm neoprene diving suit to act as bolus in their actual patient treatment setup, however their patient still experienced grade 4 myelosuppression.

Another concern with patients receiving significant bone marrow radiation dose would be secondary malignancies such as leukemia and myelodysplastic syndrome. This has been reported in patients receiving a mean bone marrow dose of 5.2 Gy when treated with total body irradiation and chemotherapy for non-Hodgkin lymphoma [14].

Disease response was moderate in our two treated cases with each experiencing a partial response immediately following treatment but soon after relapsing. Prior success had been shown with TSEBT to a dose of 12 Gy with a complete response in 88%, but often repeat total skin treatment may be needed at these low doses [7].

Although TSHT allows for improvements in dose homogeneity when compared to TSEBT, these cases illustrate the need for further research on the safety of TSHT, specifically in the case of bone marrow toxicity. We attempted to avoid bone marrow toxicity by limiting the mean dose to 2 Gy. Yet, significant myelosuppression was still encountered. Caution should be utilized when using TSHT as a therapy for mycosis fungoides. Further, patients will likely benefit from enrollment on various approaches to total skin especially in the context of TSHT. Patients who have been previously treated or are currently being treated with chemotherapy that have a low hematopoietic reserve may be especially at risk. Currently, clinical trials are underway to examine patients who will receive TSHT for treatment resistant cutaneous lymphoma [15]. These results will be a welcome addition to the literature and may help clarify safety and tolerability of this treatment approach.

## Abbreviations

HT: Helical tomotherapy
OAR: Organs at risk
PRBC: Packed red blood cell
TBI: Total body irradiation
TSEBT: Total skin electron beam therapy
TSHT: Total skin helical tomotherapy
